# Hippocampal Neuro-Networks and Dendritic Spine Perturbations in Epileptogenesis Are Attenuated by Neuroprotectin D1

**DOI:** 10.1371/journal.pone.0116543

**Published:** 2015-01-24

**Authors:** Alberto E. Musto, Chelsey P. Walker, Nicos A. Petasis, Nicolas G. Bazan

**Affiliations:** 1 Neuroscience Center of Excellence, Louisiana State University Health Sciences Center, New Orleans, Louisiana, United States of America; 2 Department of Chemistry, Loker Hydrocarbon Research Institute, University of Southern California, Los Angeles, California, United States of America; Royal College of Surgeons in Ireland, IRELAND

## Abstract

**Purpose:**

Limbic epileptogenesis triggers molecular and cellular events that foster the establishment of aberrant neuronal networks that, in turn, contribute to temporal lobe epilepsy (TLE). Here we have examined hippocampal neuronal network activities in the pilocarpine post-status epilepticus model of limbic epileptogenesis and asked whether or not the docosahexaenoic acid (DHA)-derived lipid mediator, neuroprotectin D1 (NPD1), modulates epileptogenesis.

**Methods:**

Status epilepticus (SE) was induced by intraperitoneal administration of pilocarpine in adult male C57BL/6 mice. To evaluate simultaneous hippocampal neuronal networks, local field potentials were recorded from multi-microelectrode arrays (silicon probe) chronically implanted in the dorsal hippocampus. NPD1 (570 μg/kg) or vehicle was administered intraperitoneally daily for five consecutive days 24 hours after termination of SE. Seizures and epileptiform activity were analyzed in freely-moving control and treated mice during epileptogenesis and epileptic periods. Then hippocampal dendritic spines were evaluated using Golgi-staining.

**Results:**

We found brief spontaneous microepileptiform activity with high amplitudes in the CA1 pyramidal and stratum radiatum in epileptogenesis. These aberrant activities were attenuated following systemic NPD1 administration, with concomitant hippocampal dendritic spine protection. Moreover, NPD1 treatment led to a reduction in spontaneous recurrent seizures.

**Conclusions:**

Our results indicate that NPD1 displays neuroprotective bioactivity on the hippocampal neuronal network ensemble that mediates aberrant circuit activity during epileptogenesis. Insight into the molecular signaling mediated by neuroprotective bioactivity of NPD1 on neuronal network dysfunction may contribute to the development of anti-epileptogenic therapeutic strategies.

## Introduction

Temporal lobe epilepsy (TLE) or limbic epilepsy is a common adult epileptic disorder characterized by spontaneous recurrent seizures that arise from the hippocampus and other limbic structures, and may propagate to other brain regions, triggering secondary severe generalized seizures [1]. Aside from neurosurgical resection, which benefits only a small population of TLE patients [2], there are no other effective treatments or preventive strategies for TLE [3].

Development of TLE or limbic epileptogenesis [4, 5] involves a partially-understood molecular cascade [6] that results in aberrant neuronal connectivity [7, 8] from a multi-architecture neuronal network of the limbic system connected to hippocampal formation [9] that contributes to a systemic process in the brain that leads to chronic recurrent spontaneous seizures [10]. Unfortunately, there is no current effective treatment that prevents, or a disease-modifying therapy for epileptogenesis. Although, there is an abundance of diverse antiepileptic drugs, most of them exert their actions by inhibition of the voltage-gated sodium or calcium channels or by potentiation of the GABA-mediated synaptic transmission [11], which results in a symptomatic approach in most cases; the goal of these drugs is to eliminate or at least reduce the number and/or severity of seizures. However, the molecular and cellular data regarding epileptogenesis indicate a wide spectrum of potential treatment targets.

Docosahexaenoic acid (DHA), an omega-3 essential fatty acid family member, is concentrated and avidly retained in phospholipids of synaptic and other neural membranes [12, 13]. Recent evidence suggests that omega-3 essential fatty acid improves neurological outcomes in models of epilepsy [14, 15] and is neuroprotective [12, 16, 17].

DHA-derived docosanoids mediate neuroprotective bioactivity [18]. The stereoselective mediator neuroprotectin D1 (NPD1; 10*R*,17*S*-dihydroxy-docosa-4*Z*,7*Z*,11*E*,13*E*,15*Z*,19*Z*-hexaenoic acid) has been shown to attenuate damage resulting from ischemia-reperfusion [19]. NPD1 promotes cell survival and homeostasis regulation during cellular damage [18]. Hippocampal NPD1 pool size increases after seizures [15]. Moreover, seizure progression severity and hippocampal hyperexcitability are attenuated upon intraventricular administration of NPD1 [15]. Therefore, NPD1-molecular signaling could modulate neuronal circuitry-modified mechanisms in epileptogenesis.

Here we examined the neuroprotective bioactivity of NPD1 on limbic neuronal network activities in the pilocarpine model of TLE [20] in freely-moving mice using dorsal hippocampal-implanted multi-microelectrode arrays [21, 22]. We observed that NPD1 reduces brief spontaneous epileptiform field potentials and dendritic spine loss in the dorsal hippocampus, with resultant limited hippocampal epileptic activity. Understanding the cell-type-specific molecular and cellular events underlying NPD1 bioactivity during limbic epileptogenesis will contribute to development of improved therapies for counteracting neuronal network dysfunctions, thus limiting TLE.

## Methods

### In Vivo Model of Limbic Epileptogenesis

Status epilepticus (SE) was induced by a single dose of pilocarpine hydrochloride (250 mg/kg) (Sigma Aldrich, St. Louis, MO) administered intraperitoneally (*i.p.*) 30 minutes after methyl scopolamine nitrate (1 mg/kg; *i.p.*, Sigma Aldrich) in 52 C57BL/6 adult male mice (20–25g) (Charles River Labs, Wilmington, MA) [15]. Animals were placed in individual cages and monitored during and after SE, and seizures were rated [23]. Non-intermittent seizure activity, stages 3 and/or 4 [24, 25], for each mouse was limited to 90 minutes using a single dose of diazepam (10 mg/kg, *i.p.*, Sigma Aldrich). Then each animal was monitored by trained laboratory personnel in a temperature-controlled surgical room until full locomotor recovery was observed (2–4 hours).

**Figure 1:**
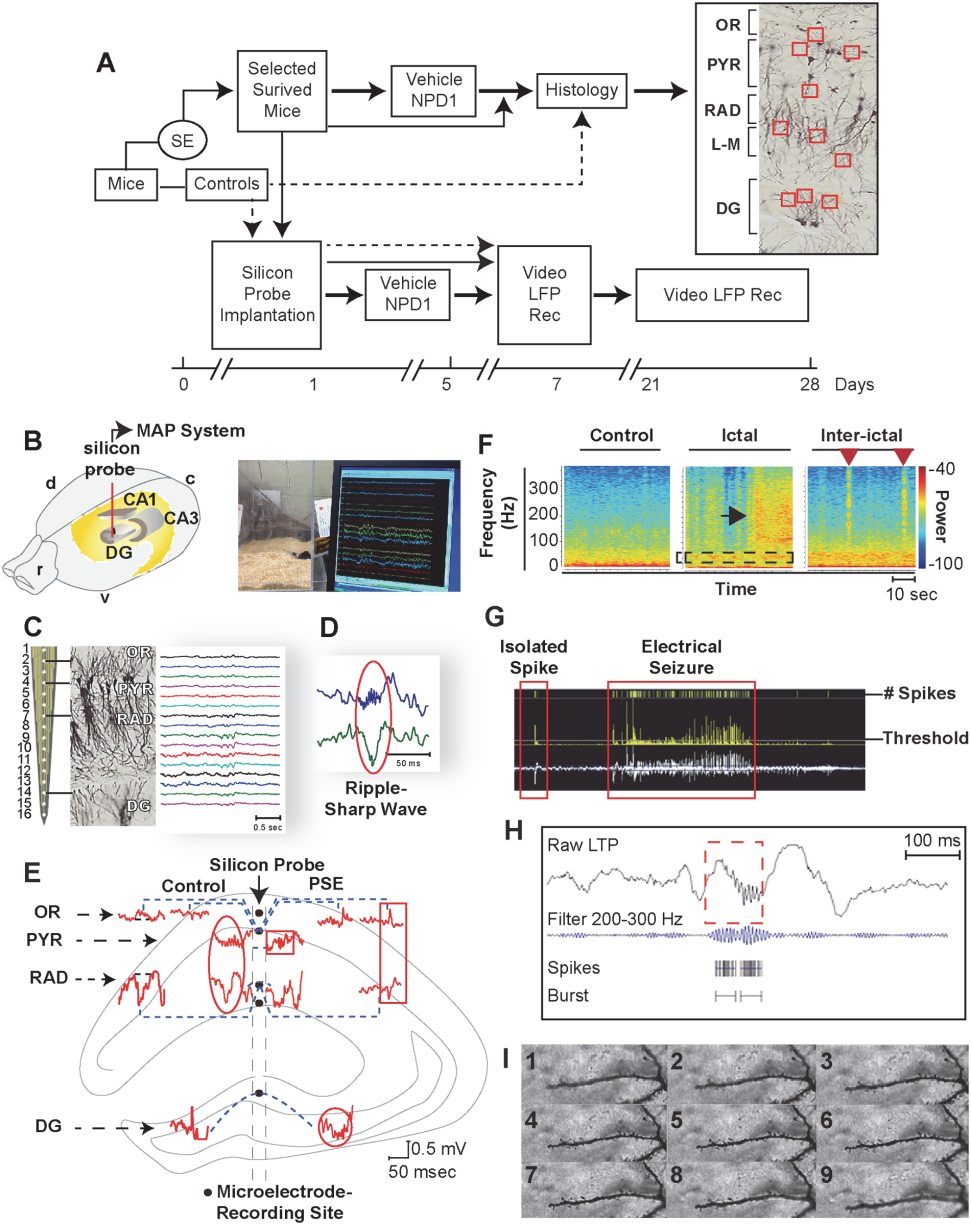
Experimental design to evaluate hippocampal aberrant neuronal networks in an epileptogenesis-induced post-status epilepticus model in mice. A: Algorithm depicting experimental design. Dashed line: control mice; thin line: mice in epileptogenesis non-treatment group; bold line: mice in epileptogenesis or epilepsy groups treated with NPD1 or vehicle. Right: Area of interest for dendritic spine analysis in hippocampal layers (Stratum oriens, SO; Stratum Lacunosum-Moleculare, L-M; and the DG). B: Silicon probe (red line) implanted in dorsal hippocampus. r: rostral; v: ventral; c: caudal; d: dorsal. Below: Representative simultaneous behavior and local field potential (LFP) recordings in freely-moving mice. LFPs were recorded from a silicon probe implanted in the dorsal CA1-dentate gyrus (DG) axis using Multiple Acquisition Processor (MAP) data acquisition system (Plexon). C: Silicon probe with 16 channels in the dorsal hippocampus, showing projections from CA1 pyramidal and granular cells from the DG using Golgi staining and hippocampal layers: stratum oriens (OR), pyramidal layer (PYR), stratum radiatum (RAD), and DG and representative LFPs (right traces). D: Representative ripple-sharp wave complex (circle) from CA1. E: Spontaneous LFP patterns from dorsal hippocampal regions (graphic in the background) of adult mice recorded from silicon probe before (control) and 14 days after SE (PSE) induced by pilocarpine administration. Control: Positive wave in OR associated with negative sharp wave in RAD, dentate spikes in DG, and ripples associated with sharp wave (oval). In PSE: fast ripple (square box); high frequency oscillation (HFO) in DG (circle) associated with positive spike in OR and interictal spikes with opposite phase in CA1 (rectangle). F: Seizures show a high frequency component (black arrow) preceded by an increase in gamma band (box), a reduced theta band. Interictal epileptiform activity with high frequency component (red arrows). G: Screenshot of automatic detection and quantification of spikes from seizures and a burst of HFO (H) using Offline Sorter V3 (Plexon). Rec: recording. I: Representative pictures (1–9) of one dendrite captured using the 100x/oil high magnification objective using Z-stack method of imaging (233–260 frames for each dendrites) at 0.3 μm between each consecutive image.

Then surviving animals (n = 45, 60%) were randomized and placed in individual cages until surgery and/or further treatment (Fig. 1A). Control animals (n = 10) received methyl scopolamine. Each mouse was maintained in an individual cage at a constant temperature with an artificial 12-hour light/dark cycle with access to food and water *ad libitum*. Studies were performed according to National Institutes of Health and ARRIVE guidelines and in accordance with nationally accepted principles in the care and use of experimental animals. The Institutional Animal Care and Use Committee (IACUC) at the Louisiana State University Health Sciences Center (LSUHSC), New Orleans, approved the animal protocols used for this study.

### Local Field Potentials and Seizures Recording and Analysis

Mice that survived 24 hours following SE were selected randomly and a silicon probe with 16 electrodes (spacing 100 μm, NeuroNexus, Ann Arbor, MI) was implanted in the dorsal hippocampus (from the bregma: 1.8 mm posterior; 1.5 mm lateral and 2.5 mm depth). Briefly, each mouse was placed under anesthesia induced by a mixture of ketamine (200 mg/kg) and xylazine (10 mg/kg) (Vedco Inc., Saint Joseph, MO) (Fig. 1B). During surgery, the probe was placed in the superficial layers of the cortex and then moved inward, slowly guided by stereotaxic equipment (Kopf Instruments, Tujunga, CA), using a surgical microscope and sterilized neurosurgical instruments. The resulting hole was covered by Surgicel (Ethicon Inc., San Angelo, TX) and saturated with a sterile cerebral spinal fluid (Harvard Apparatus). A stainless steel screw (Plastics One, Roanoke, VA) was implanted in the occipital bone as a ground wire for the silicon probe. Plastics One gel was used to attach the probe and screw it to the skull. After recovery from anesthesia, the mice were placed in sterilized individual transparent polyacrylic glass cages with gel food (Nutra-Gel Diet, Bio-Serv, VWR International, Inc., West Chester, PA) provided *ad libitum* in a room with a 12-hour light/dark cycle per day. Laboratory and veterinarian personnel monitored the mice according to approved IACUC protocols, and mice that showed impaired locomotor activity, grasping or a pinch reflex, were excluded from the study (n = 4). For euthanasia, animals were deeply anesthetized with ketamine hydrochloride and xylazine (200 mg/kg + 10 mg/kg; i.p.) prior to death by decapitation.

For local field potential (LFP) recordings, pre-amplified headstages (16 HST; Plexon, Dallas, TX) were connected to the probe and then local field potentials were amplified (1000x), band-pass filtered (0.1–300 Hz), and digitalized at 1 KHz using a MAP data acquisition system (Plexon, Dallas, TX). Then continuous LFP activity (4–5 minutes) from each freely-moving mouse, placed in separate Plexiglas chambers, was recorded and sampled (10–12 samples/hour) every 5 minutes from 10:00 a.m. to 4:00 p.m. using a MAP (Plexon) and video-recorder system (Sony Handycam) (Fig. 1B); this was conducted on day 7 and from days 21 to 28 after SE. Then the number of spontaneous motor seizures (stage 3 or and 4), according to the Racine’s score [25], and their durations were quantified each day (5–6 hours) by an investigator blinded to the treatment, and then the total number of those seizures was summarized for each animal. To evaluate epileptogenesis as a consequence of SE, only treated animals that had LFP recordings one week after SE (epileptogenesis), were included in the analysis at days 21 to 28 after SE (epilepsy).

**Figure 2:**
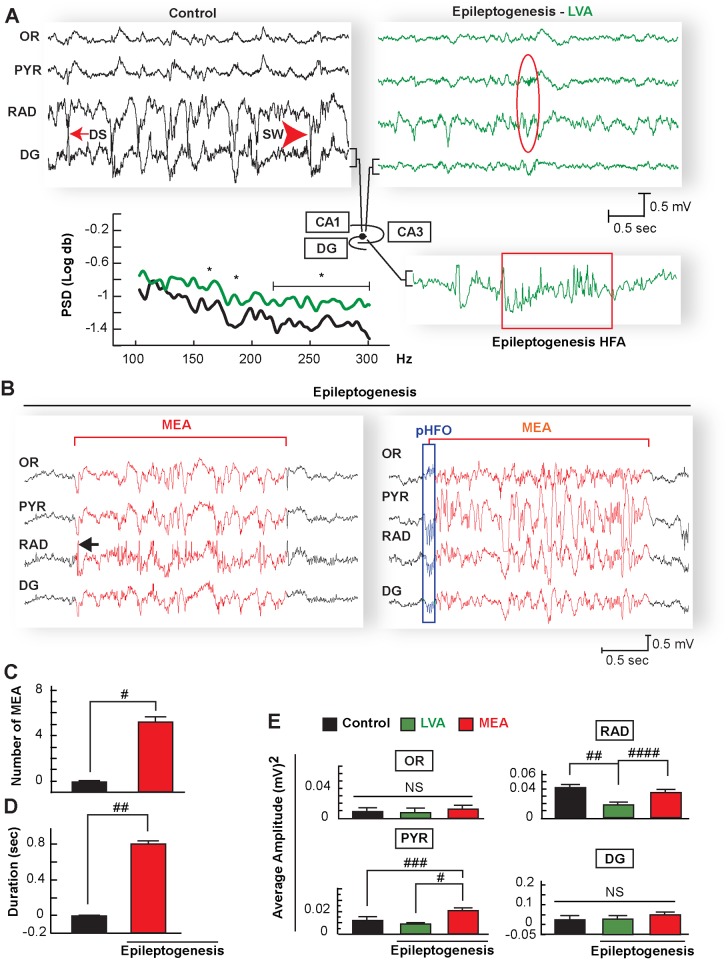
Hippocampal micro-epileptiform activities with high amplitude in the CA1 pyramidal-stratum radiatum region during epileptogenesis. A: Comparative local field potential (LFP) recordings of stratum oriens (OR), pyramidal layer (PYR), stratum radiatum (RAD), and dentate gyrus (DG) in the dorsal hippocampus during spontaneous immobility. Left is from a naïve mouse (control), which displays physiological electrical morphology of dentate spikes (DS) and sharp waves (SW), in contrast with a mouse in epileptogenesis (right) showing global low voltage activity (LVA) and ripples-sharp wave (red oval) associated with a marked reduction of amplitude in RAD. Below left: High frequencies activity (HFA) from DG at 150, 170 and above 215 are increased during LVA from mice in epileptogenesis (n = 7) compared with control mice (n = 7). Below right: representative trace from DG during LVA showing high frequency activity (box). Compare traces signed by brackets from control, epileptogenesis-LVA and epileptogenesis-HFA. PSD: Power spectral density from 100 to 300 Hz band, sec = seconds; mv = millivolts. * = p<0.05, One-way ANOVA); B: Representative micro-epileptiform activity (MEA, red traces) emerging from a LVA (black traces) in OR, PYR, RAD, and DG in epileptogenesis. Note MEA in hippocampal CA1 is characterized by successive spikes after a high amplitude fast spike (arrows) in RAD or initiated by a generalized burst of pathological HFO (pHFO, blue rectangle) followed by a high amplitude of synchronized spikes. Number (C) and duration (D) of micro-epileptiform activity (MEA) increases in mice during epileptogenesis (N = 7) compared with control mice (N = 7) #: p = 0.0001 ##: p = 0.001 two sample t-test. E: Average of square amplitude of LFP samples (n = 17 each group) of each hippocampal layer from periods of immobility in control mice (N = 7) and mice undergoing epileptogenesis (N = 7) during LVA and MEA periods. Note that MEA has a high amplitude in the pyramidal layer (PYR) and stratum radiatum (RAD) compared with LVA and control. Meanwhile LVA amplitude is reduced in RAD compared with control and MEA. Bars indicate means and error bars represent S.E.M.; #p = 0.0001; ##; p = 0.001, ###: p = 0.002 ####; p = 0.007, NS: no significant; one-way ANOVA. sec = seconds; mv = millivolts. OR: stratum oriens, DG: dentate gyrus.

Each channel for each mouse was inspected using 1D window of Neuroexplorer (Nex Tech, TX) and with reference to the video recordings and hippocampal field oscillatory activities. Voltage-versus-depth profiles, ripples associated with sharp waves, or epileptic patterns (Fig. 1C–E) were selected using timestamp functions and after the following analysis: Band frequencies for delta (0.1–3.9 Hz), theta (4–8 Hz), beta (13–20 Hz), low gamma (21–40 Hz), and bands from 100–300 Hz were selected and quantified using power spectral density. Briefly, signal values were multiplied by the coefficients of the Hann window, and discrete fast Fourier transformations of the results were calculated using formulas defined previously [26] and then normalized using Neuroexplorer (Nex Technologies, Madison, AL). Then delta epochs (5–6 seconds each) (Fig. 2A) from each local field potential were determined by periods of immobility, without artifacts or noise, selected by an investigator blinded to the treatment, and confirmed by reproduction of video recordings and by calculating the ratio of delta and theta frequency bands in the CA1 hippocampal region [27].

Amplitude of the LFP, representative of each hippocampal layer, was determined, converted into a rate histogram (amplitude vs. time; bin = 0.01sec), and exported to a Microsoft Excel format using Neuroexplorer (Nex Technologies, Madison, AL); then the square was calculated. The number of samples and their duration between vehicle and NPD1-treated animals were not statistically significance (p = 0.2; p = 0.3 respectively).

Microepileptiform activity (MEA) was displayed as electrographic seizure-like discharges from each delta epoch (Fig. 2B). These events were identified and separated from normal activity based on paroxysmal changes arising from the background with energy of the signal above 4–5 standard deviation (SD), power above 100Hz, no more than 4 sec of duration with periodic bursts of spikes synchronized in more than 4 contiguous channels (400 μm-500 μm of hippocampal area). Then the spikes from filtered LFPs (200 to 300Hz) were selected using a threshold function above 4–5 SD from the mean baseline signal, and the number and duration of those events were automatically quantified using NeuroExplorer (Plexon Inc., Dallas, TX) and Offline Sorter (Plexon Inc., Dallas, TX) (Fig. 1F–H) [26].

Spontaneous electrographic seizures were identified from LFP background by high amplitude spike activity above 4 SD from the baseline with a frequency above 4 Hz progressive frequency of spiking activity for a minimum of 10 seconds [28], and confirmed by spectrogram analysis (Fig. 1F). These types of discharges were associated with partial seizures or tonic-clonic seizures. Then the total number of epileptic spikes above the threshold were quantified automatically using Offline Sorter software (Plexon Inc., Dallas, TX).

Spontaneous isolated spikes were observed in all channels with frequency band of 96Hz–107Hz, with >50 millivolts of amplitude and <10 milliseconds of duration. Then LFPs were filtered using those parameters, and these spikes were quantified automatically using Offline Sorter software.

High frequency oscillatory bursts were identified from those delta epochs automatically [26] using multiple trains of spikes that marked high-frequency activity (>200Hz), then burst analysis groups of spikes (4 SD above baseline) where automatically quantified (Fig. 1H).

Mice that presented excessive noise or artifacts in their LFP recordings were excluded from the study. Artifacts such as head movement or grooming were excluded by visual inspection of video-LFP recordings. At the end of the experiment, histological verification of probe placement in the dorsal hippocampus was confirmed by histology [29] for anatomic-physiological correlation of different hippocampal layers [21].

### In Vivo NPD1 or Vehicle Treatment

NPD1 and vehicle solutions were prepared according to previous protocols [30], and then NPD1 (570 μg/kg) or vehicle was administered *i.p.* once a day for five consecutive days 24 hours after termination of SE.

### Histology and Analysis of Dendritic Spines

For histology analysis, control mice and mice undergoing epileptogenesis, which included mice treated with NPD1 or vehicle, were deeply anesthetized [29] 7 days post-SE, and brains were dissected and processed using an FD Rapid GolgiStain Kit following the manufacturer’s instructions (FD Neurotechnologies, Inc., Columbia, MD). Coronal sections (80 μm) were made and then mounted, air-dried, dehydrated in alcohol, cleared in xylene and coverslipped. Then dorsal hippocampal sections were analyzed using a bright light deconvolution microscope (Axiovision Zeiss, USA), and individual Golgi-impregnated principal cells were identified at 40x field. Dendrites from the stratums oriens (SO), stratum radiatum (RAD), and lacunosum-moleculare layers (L-M) of the CA1 and outer molecular layer (OM) from the dentate gyrus (DG) were captured using the 100x/oil high magnification objective and the Z-stack method of imaging (233–260 frames for each dendrites). Z-stacks were taken with a step size of 0.3 μm between each consecutive image. Analysis of the number of dendritic spines was done using Image J software (National Institute of Health) by investigators blinded to the experimental conditions. Briefly, dendritic segments (10 μ mm in length) were identified from two-dimensional image stacks selected from all frames, then individual dendritic protrusions were tracked manually along dendrites. The serial set of frames (Fig. 1I) allowed for identification of individual dendritic protrusions. Then the number of dendritic spines (irrespective of their morphological characteristics that emerged perpendicular from the dendritic shaft) for each segment of the dendrite (10 μm) was quantified according to established procedures using Image J software (National Institute of Health) [31]. Images were recorded using an Axioplan 2 microscope (Carl Zeiss Inc., Thornwood, NY) coupled with AxioCam and Axiovision software (Carl Zeiss Inc.). We then calculated the number of dendritic spines per segment of individual dendrites per hippocampal subfield for each treated group of animals. Each group had an equal number of analyzed dendrites.

### Statistics

The data retrieved from each experiment were averaged and expressed as means ± S.E.M. For statistical significance, one time point (or more than two observations) was analyzed using two-sample Student’s *t*-Test and ANOVA following post-hoc tests (Tukey-Kramer and Hsu’MCB). Cumulative probability plot was calculated and the log-rank test was used to determine statistical differences. A *p*-value was placed in each result. All data analysis was conducted using JMP 8.0 statistical software from SAS (Cary, NC) [32, 33].

## Results

**Figure 3:**
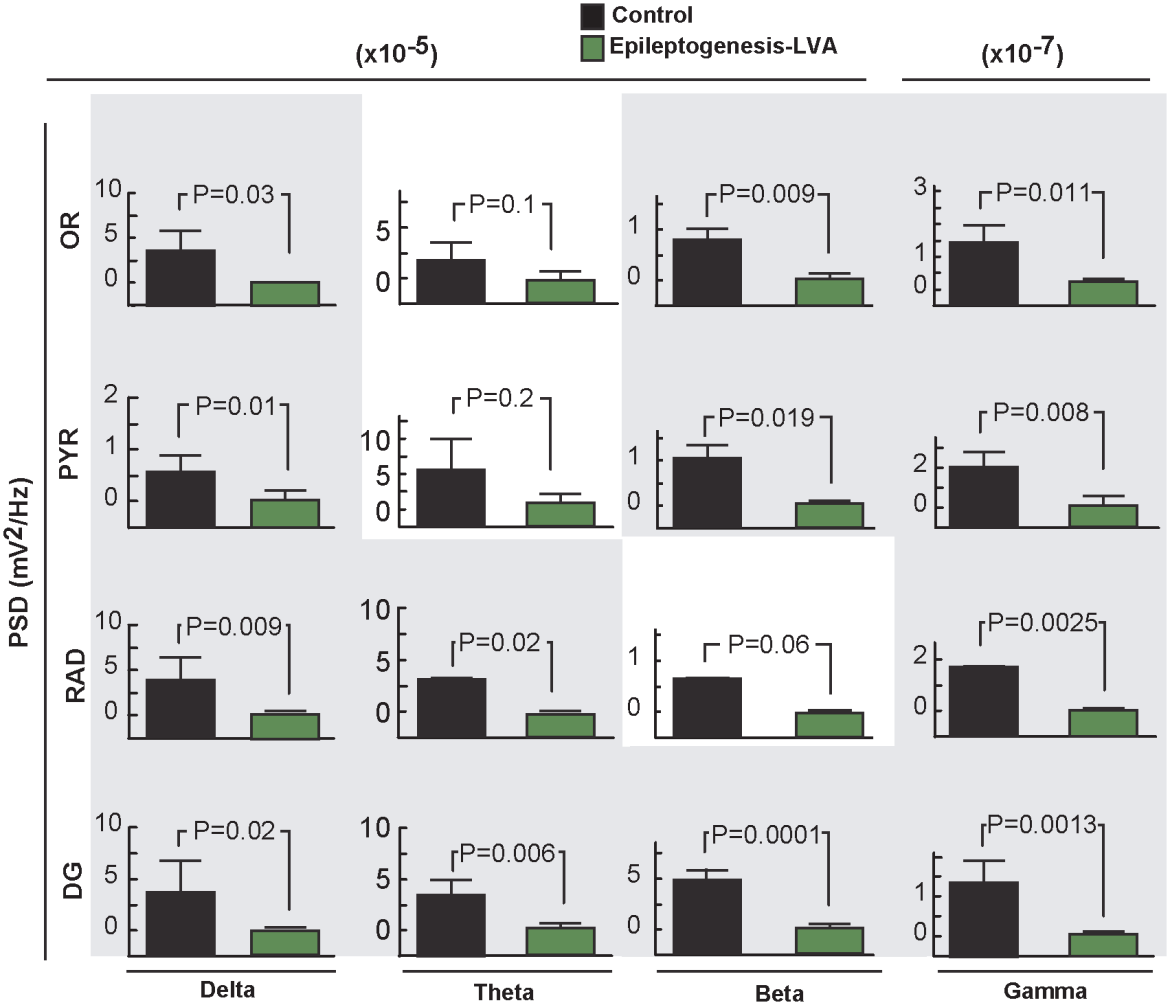
Frequency analysis of hippocampal layers in epileptogenesis. A: Analysis of frequency bands (delta, theta, beta and gamma) from different hippocampal layers during period of immobility in control (n = 7) and in epileptogenesis mice (n = 7). All frequency bands are reduced in epileptogenesis, except for theta in OR and PYR layers. Bars indicate means, and error bars represent S.E.M.; P: p values, two sample t-test. Power spectral density, PSD.

We observed, in agreement with Buzsáki and colleagues [21], physiological patterns in hippocampal layers that included deep-voltage profiles, the ripple sharp wave complex, and DG spikes in control animals, allowing for recognition of neuronal network assemblies (Fig. 1C–E, Fig. 2A) relevant to hippocampal function [21] and inter-ictal or seizure state. At seven days after SE and during periods of immobility in freely-moving mice, although convulsive seizures were not observed (Racine’s score > 3), consistent with previous observations [34, 35] the dorsal hippocampus displayed disruptions in electrical patterns, mainly in all hippocampal layers (Fig. 2A). The ripples were associated with a marked reduction of amplitude in the hippocampal CA1 RAD (p = 0.002 vs. control mice), which attenuates sharp wave profiles (Fig. 2A). Also, the DG field potential showed a lack of dentate spikes associated with an increase in signal power at frequencies above 150 Hz (p<0.05 vs. control mice) (Fig. 2A) that persisted three weeks after SE. In addition, attenuation of low frequency bands (i.e., delta, theta, beta and gamma) were present in all hippocampal layers except theta, in the SO and PYR (Fig. 3). Since voltage reduction was predominant during this post-status epileptic period, we denoted this electrical pattern as low voltage activity (LVA), reflecting a global neuronal network disruption within the hippocampus (Fig. 2A, Fig. 3).

**Figure 4:**
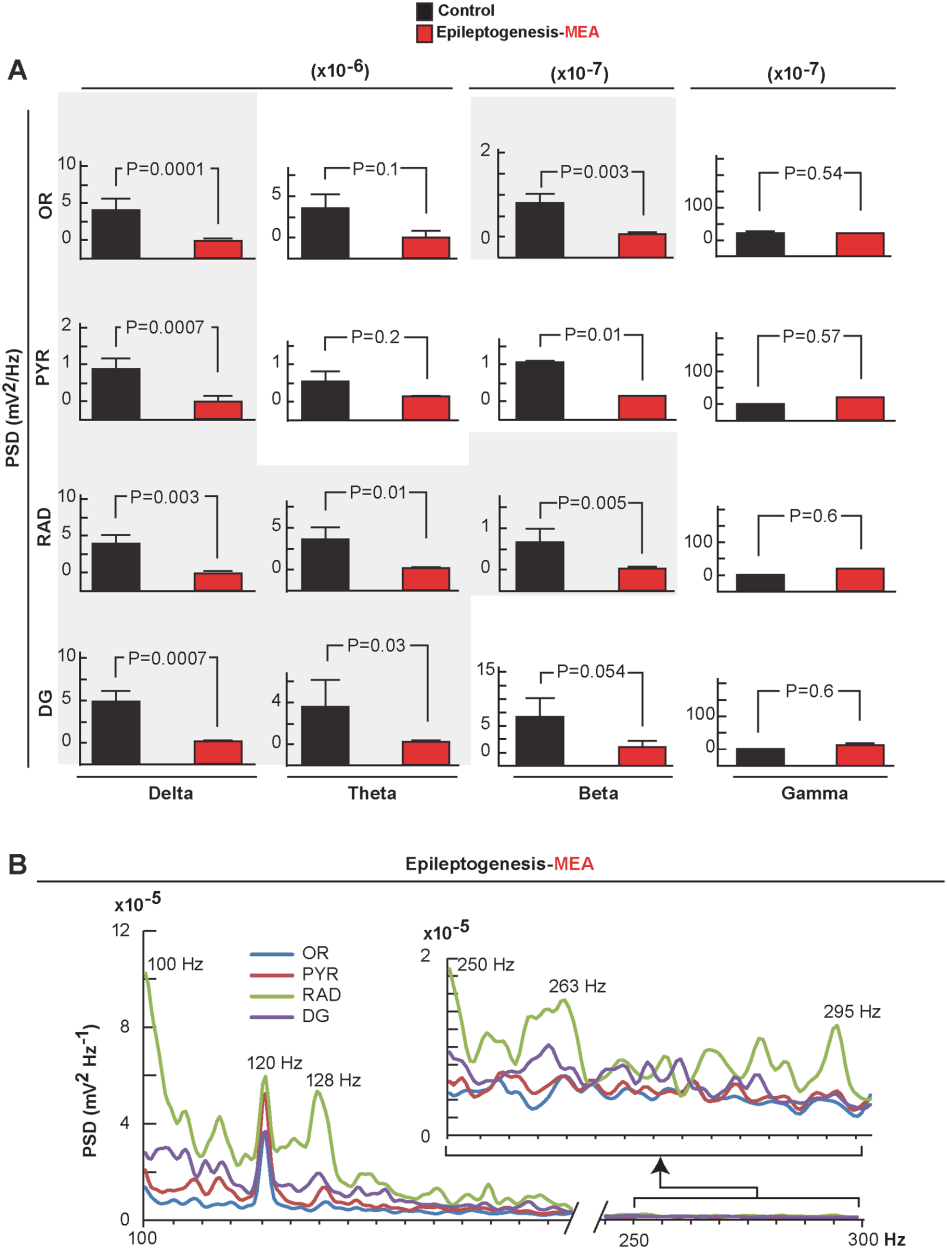
Frequency analysis of microepileptiform activity in epileptogenesis. Frequency bands (delta, theta, beta and gamma) show reduced microepileptiform activity (MEA), except for theta and gamma, in all hippocampal layers, and reduced beta activity in the stratum oriens (OR) and dentate gyrus (DG) compared with controls. Bars indicate means and error bars represent S.E.M. P = p value (two sample t-test). B: Average of values of the frequency power of the signal of MEA (n = 7) showing peaks above 120 Hz synchronized in all hippocampal layers. Stratum radiatum, RAD; pyramidal layer, PYR; power spectral density, PSD.

LVA was interrupted by an unexpected brief spontaneous micro-epileptiform activity (MEA) (Fig. 2B). Although fluctuations in amplitude were present in neuronal circuitry, these type of discharges were not observed in control animals (Fig. 2C and D). MEA was characterized by successive bursts of spikes with high amplitudes (Fig. 2B), mainly in the PYR and RAD (P = 0.007 vs. LVA) (Fig. 2E) simultaneously in all hippocampal layers (4.6 ± 1.06 events/per delta epoch; with duration of 0.8 sec ± 0.14 sec) (Fig. 2 B–D). These MEA showed reduction of the power in the low frequency bands, especially in delta and beta in the OR and DG, compared with controls (Fig. 4A); a trend of increased gamma waves occurred in all layers observed. Also, within the MEA, high frequency activity was present associated with a peak of 120 Hz in all hippocampal layers and a trend of higher frequency bands in the RAD (Fig. 4B). In addition to different patterns from MEA, spontaneous isolated spikes were observed in only 66% of mice after SE; however these were not statistically different from controls that showed similar spike profiles (P = 0.09 vs. control) (S1 Fig.).

**Figure 5:**
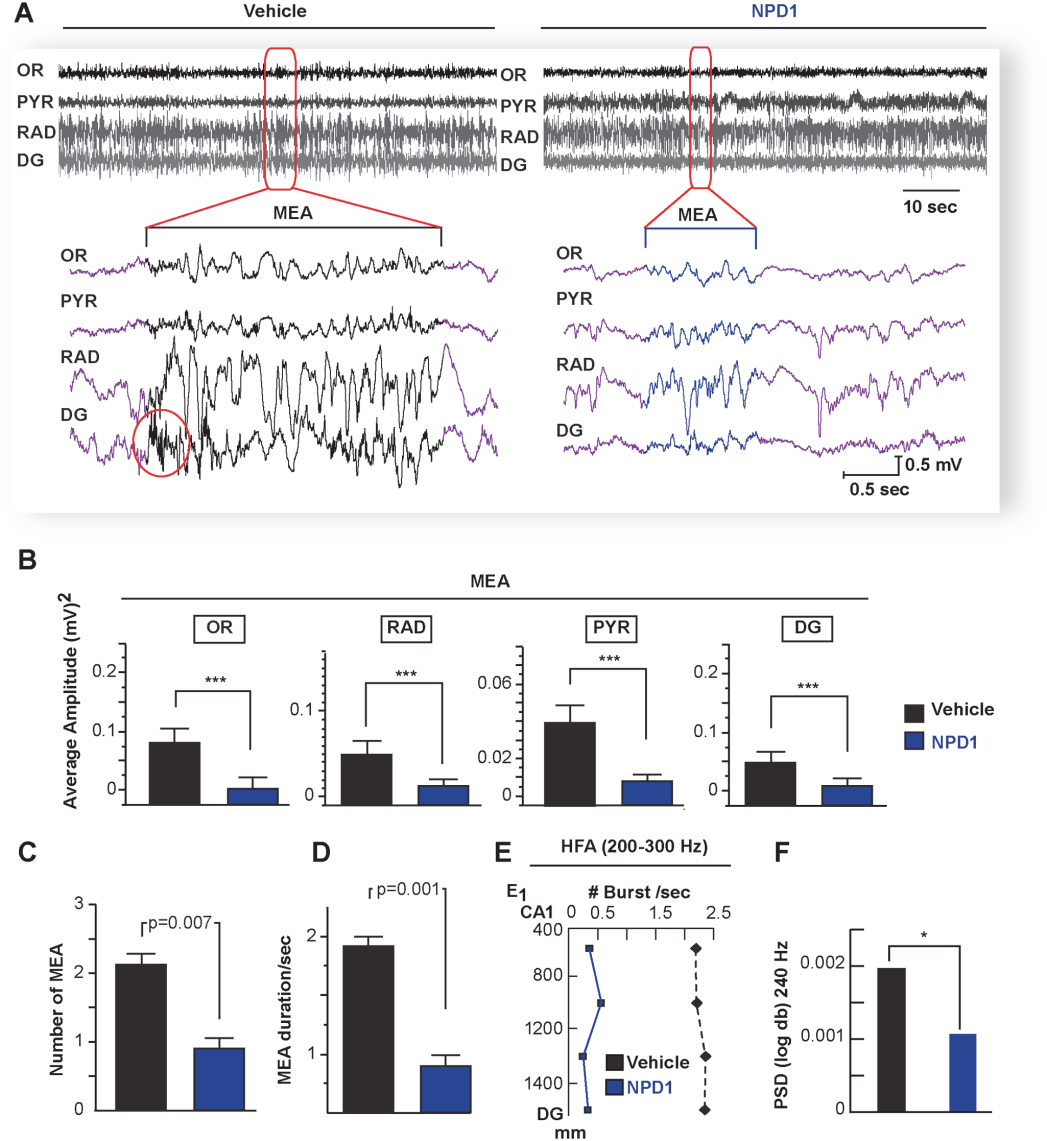
Administration of neuroprotectin D1 during epileptogenesis attenuates micro-epileptiform activity. A: Hippocampal local field potentials from neuroprotectin D1 (NPD1)- and vehicle-treated mice (vehicle) one-week post-status epilepticus (epileptogenesis). Note NPD1-treated mice display attenuation of micro-epileptiform activity (MEA, below brackets) and reduction of the occurrence high frequency oscillation burst (circle) present in vehicle-treated mice. B: Averaged amplitude of MEA is reduced in all hippocampal layers in NPD1-treated mice (n = 7) compared with vehicle-treated mice (n = 7). NPD1-treated mice show attenuation of the number (C) and duration (D) of MEA compared with vehicle-treated mice. E: High frequency activity (HFA) is modulated by NPD1. Note NPD1-treated mice have a reduction of bursts of hippocampal high frequency oscillations (>200Hz, E) and high frequency activity at 240 Hz (F) compared with vehicle. Bars indicate means, and error bars represent S.E.M. P = p value, two sample t-test. * = p<0.05, *** = p<0.001, OR: stratum oriens, PYR: pyramidal layer; RAD: stratum radiatum; DG: dentate gyrus.

**Figure 6:**
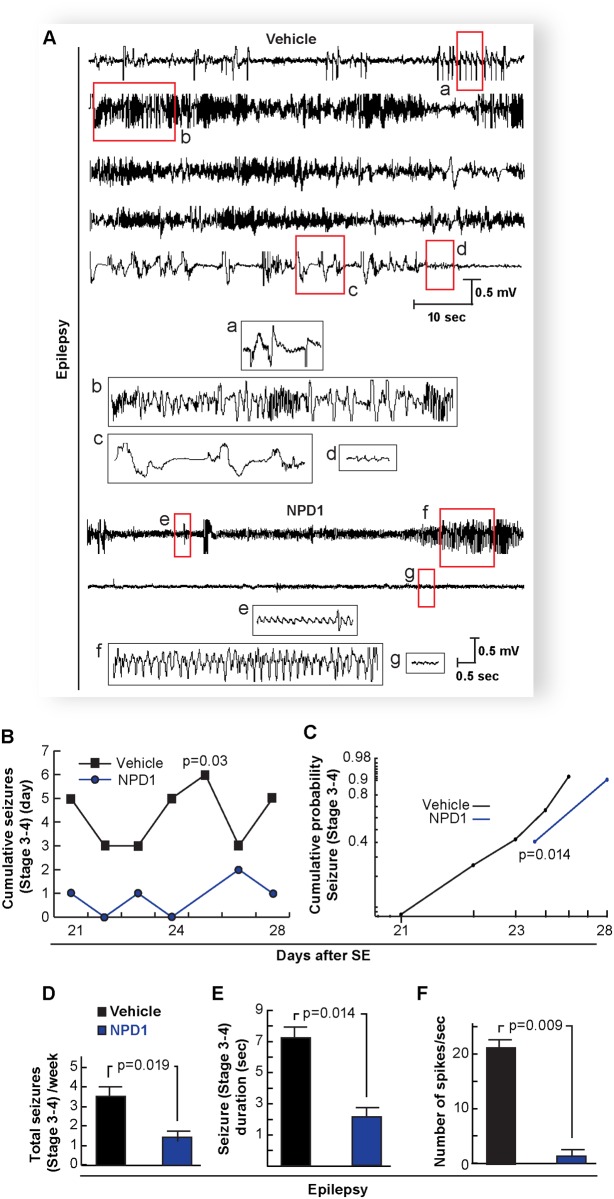
Administration of neuroprotectin D1 during epileptogenesis reduces spontaneous recurrent epileptic seizures. A: Representative local field potential from the CA1-pyramidal layer three weeks after status epilepticus (epilepsy) from mice treated with NPD1 or vehicle during 5 days after status epilepticus. Note that NPD1-treated mice (NPD1) display an attenuation of spontaneous hippocampal epileptic electrical activity compared with vehicle-treated mouse (vehicle). Insets (red boxes from a-d) are traces from the vehicle: (a) initial discharges, followed by (b) synchronized poly-spike activity interrupted by bursts of high frequency discharge; (c) spike waves; and (d) post-ictal phase. Other insets (red boxes from e-g) are traces from NPD1-treated mice: (e) initial discharge; (f) synchronized spikes; and (g) post-ictal phase. B: Cumulative generalized seizures (Racine’s score > stage 3) per day and cumulative probability (C) at the third week after status epileptics are reduced in NPD1-treated mice (n = 5) vs. vehicle-treated mice (n = 6). D: Total cumulative seizures per week and duration of each locomotor seizure (E) are limited in NPD1-treated mice compared with vehicle. F: NPD1-treated mice (n = 4) show a reduced number of epileptic spikes compared with vehicle-treated mice (n = 4). Bars indicate means, and error bars represent S.E.M. p = p value (two sample t-test).

Intra-cerebral NPD1 administration reduces seizure susceptibility in a kindling mouse model [15], and the present study discloses that systemic administration of this lipid mediator, during five consecutive days after SE (see Methods), remarkably attenuated the amplitude and number of MEA (NPD1: 0.8 ± 0.28 SEM vs. Vehicle: 2.09 ± 0.15 S.E.M.; P = 0.007) (Fig. 5A, B and C) as well as their duration (NPD1: 0.7 ± 0.07 S.E.M. vs. Vehicle: 1.72 ± 0.2 S.E.M.; P = 0.013) (Fig. 5D). Also, NPD1 reduced signal power at frequencies above 200 Hz in the DG (Fig. 5E and F). Since chronic recurrent seizures are a consequence of pilocarpine-induced SE in all adult mice [27, 34, 35], the hippocampal spontaneous epileptic seizures were analyzed during a five-day period at three weeks after SE. NPD1 administration during epileptogenesis reduced the onset of, the number, and the duration of severe spontaneous seizures compared with vehicle-treated mice (Fig. 6B–E) (Racine’s score: NPD1: 1.2 ± 0.2 S.E.M. vs. Vehicle: 3.16 ± 0.6 S.E.M., P = 0.0019; Duration: NPD1: 2.74 ± 0.72 S.E.M. vs. Vehicle: 7.23 ± 0.6 S.E.M., P = 0.014; spikes: NPD1: 1.5 ± 0.86 S.E.M. vs. Vehicle: 21.93 ± 3.23 S.E.M. P = 0.009). Also, NPD1 limited the severity of the epileptic discharge from pyramidal layer by attenuating the number of spikes compared with vehicle-treated mice (Fig. 6F).

**Figure 7:**
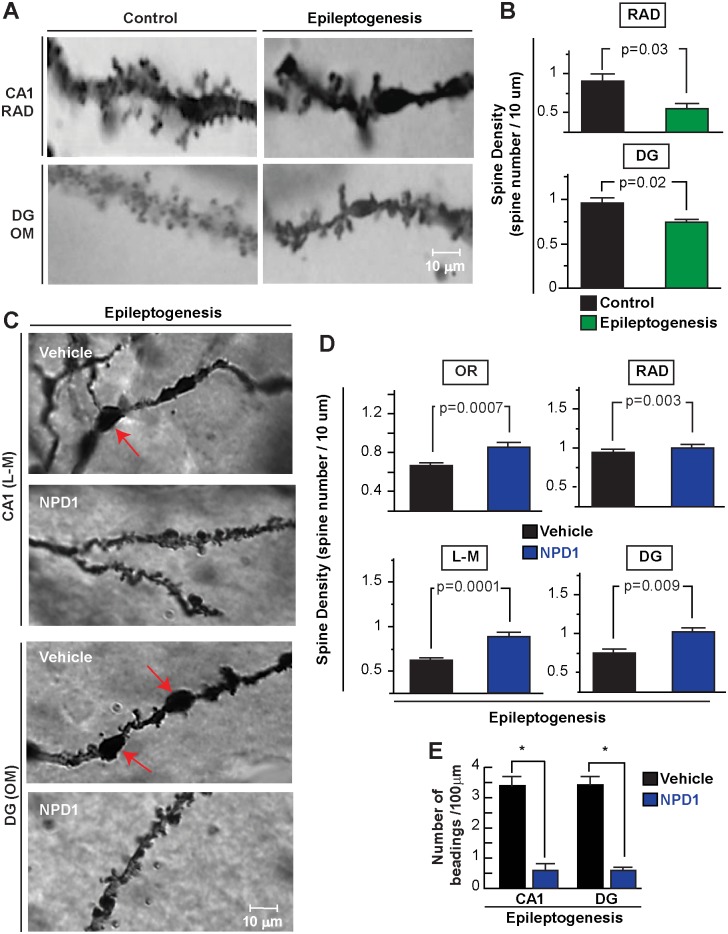
Neuroprotectin D1 induces sustained protection of dendritic spines during epileptogenesis. A: Representative dendrites from control (naïve) and a mouse at 7 days after status epilepticus (SE) from CA1, stratum radiatum (RAD), dentate gyrus (DG) and outer molecular layer (OM). B: Note loss of number of dendritic spines as a consequence of status epilepticus (PSE, n = 3) compared with control (n = 3). C: High-power light magnification of dendrite profiles of CA1 and dentate gyrus (DG) regions showing dendritic beadings in vehicle-treated mice (n = 4) (arrows) and lower dendritic spine profiles compared with NPD1-treated mice (n = 4) in epileptogenesis D: NPD1-treated mice show a higher number of dendritic spines in the hippocampal layers than vehicle. E: Number of dendritic beadings per dendrites is reduced in NPD1-treated mice compared with vehicle. Bars indicate means, and error bars represent S.E.M. p = p value. *: p<0.05, two sample t-test.

Because LFPs represent post-synaptic potential activities from synchronized neurons [21], and since dendritic spines are thought to be a morphological signature of post-synaptic sites [36] and a key target for neuronal network assemblies damaged during epilepsy [37], we asked whether MEAs were associated with hippocampal dendritic spine modification. Using Golgi staining, we observed a reduction of the number of dendritic spines per dendrite segment in CA1 and dentate gyrus regions (Fig. 7A, B, RAD: Control: 0.78 ± 0.06 S.E.M. vs. Epileptogenesis: 0.62 ± 0.03 S.E.M., p = 0.03; DG: Control: 0.82 ± 0.054 S.E.M. vs. Epileptogenesis: 0.67 ± 0.03 S.E.M., p = 0.02) after 7 days of SE. Also, dendritic swelling and beading, both of which are hallmarks of dendritic injury [38], were present as a consequence of SE. Using the same histological approach, we observed, at seven days after SE, that NPD1 administration showed higher density of spines from the dendrites of both CA1 PYR and DG-granular cells compared to vehicle-treated mice (OR: NPD1: 0.90 ± 0.04 S.E.M. vs. Vehicle: 0.69 ± 0.03 S.E.M., p = 0.0007; RAD: NPD1: 1.04 ± 0.05 S.E.M. vs. Vehicle: 0.83 ± 0.04 S.E.M. p = 0.03; L-M: NPD1: 0.93 ± 0.64 S.E.M. vs. Vehicle: 0.64 ± 0.03 S.E.M., p = 0.0001; DG: NPD1: 1.07 ± 0.05 S.E.M. vs. Vehicle: 0.80 ± 0.05 S.E.M., p = 0.009; Fig. 7 C, D) and induced less beading-like profiles (CA1: NPD1: 0.65 ± 0.16 S.E.M vs. Vehicle: 3.44 ± 0.41 S.E.M.; DG: NPD1 0.65 ± 0016 S.E.M. vs. Vehicle: 3.44± 0.39 S.E.M., p;<0.0001; Fig. 7 E).

## Discussion

The latent period between brain injury and the onset of clinical seizures offers an opportunity to evaluate anti-epileptogenic therapeutics [4, 10], to explore bioelectrical markers that can be used for early diagnostics in a population at risk, and to understand neuronal damage and axonal reorganization mechanisms in TLE [39]. The pilocarpine model of TLE in adult mice shows an onset of recurrent spontaneous seizures 7 days after SE that can continue for at least 7 weeks [27, 34, 35]. Proper description of electrical patterns of discharges, such as ripples, which reflect an specific neuronal network activity [21], or frequency analysis of neuronal activities within those electrical patterns [40, 41], may provide insight into the intrinsic neuronal circuitry involved in epileptogenesis or could be used as a biomarker of the TLE progression. We observed brief hippocampal spontaneous epileptiform activity, which we denoted as MEA, when animals were quiet or still 7 days after SE. These MEA could resemble micro-activities observed in tissue from epileptic patients [42, 43] (Fig. 2A and B). Since their amplitude was higher in the PYR and RAD, we speculated that these electrical manifestations may arise from small epileptogenic generators that could be related with strong depolarization from the CA3 associated with an increased hyper-excitability of CA1 [29], thus reflecting a complex pathological microcircuit activity in epileptogenesis. In addition, these MEA could be related to spontaneous bursts of certain groups of neuronal networks that could involve Schaffer’s collaterals before robust neuronal reorganization in epilepsy (i.e., chronic epilepsy) [44] or impairment of GABAergic perisomatic interneuronal fibers [45] that determine hippocampal network electrical activity [21], since these inhibitory fibers are damaged after SE [46]. Moreover, the MEAs described here could be some of the many diverse pathological activities present during the inter-ictal periods in TLE [29], suggesting a dynamic and complex pathophysiology for the neuronal network involved in limbic epileptogenesis.

We observed that pHFOs precede microepileptiform discharges (Fig. 2A and B), indicating an ictogenesis mechanism that could contribute to the propagation of MEA and an input of burst discharges, particularly within the pathological CA1-CA3-interconnecting micro-domain [47, 48]. Also, isolated pHFOs in the DG, as a consequence of SE (Fig. 2 A, B), may act as an endogenous “pacemaker of kindling-like mechanisms” that recruits and synchronizes other aberrant neuronal networks, which then trigger spontaneous recurrent seizures. Although the role of pHFOs as a consequence of SE or in TLE is not clear, pHFOs may attempt to halt seizures [49], thus suggesting that PYR cells present high frequency inhibitory activity for preventing the spread of epileptiform activity. However, this inhibitory activity might be overwhelmed, resulting in failed seizure inhibition [50].

Previously, we observed that hippocampal hyperexcitability, followed by electrical stimulation in the CA1 region, is attenuated by NPD1 [15]. After intra-ventricular administration of NPD1 during kindling epileptogenesis, we observed that NPD1 limits progression of seizure severity and hippocampal hyper-excitability, including abnormal patterns of the electrical seizures [15], suggesting that NPD1 modulates the neuronal network in the CA1 during ictogenesis, which is critical in kindling epileptogenesis [15] and in human and experimental TLE [48]. Hippocampal hyper-excitability represents a paroxysmal and self-limited neuronal network phenomenon characterized by the hyper-synchrony of a large population of neurons [51] that could be initiated by bursts of pHFO and modulated by NPD1 resulting in decreased MEA (Fig. 5). However, the precise neurotransmission mechanism mediated by NPD1 in epileptogenesis remains to be further defined.

Epileptiform activity during initiation of epileptic discharges in the neocortex [52] suggests enhanced postsynaptic activity [53]. The aberrant neuronal network activity observed here from LFP recordings may reflect the linearly-summed postsynaptic potentials from small populations of principal cells [21, 53] that takes place in dendritic spines [34, 54] that survive after SE. Dendritic spine reorganization in the DG occurs during seizure susceptibility, development and spontaneous recurrent seizures [44]. Moreover, dendritic spine rearrangement could result from seizure-induced dendritic spine loss [55]. Although the mechanism of NPD1 on neuronal network activity is not clear, we showed that NPD1 could provide protection for dendrites and dendritic spines during epileptogenesis, which reached up to a similar number of dendrites for animals from the control group (S2 Fig.: CA1 L-M: NPD1 1 ± 0.08 S.E.M. vs. Control: 0.82 ± 0.16 S.E.M., p: 0.2; DG: NPD1: 1.18 ± 0.10 S.E.M. vs. Control: 1.14 ± 0.11 S.E.M., p: 0.7), and thus could prevent aberrant connectivity in the hippocampus. Therefore, NPD1 administration could also initiate or strengthen endogenous neuroprotective signaling in dendritic spines and limit their damage.

NPD1 modulates neuroinflammatory signaling and reduces oxidative stress-induced apoptosis by modulating pro-inflammatory gene expression and the Bcl-2 family of proteins [18, 56]. Also, since BDNF, NGF, NT3 and other neurotrophins are NPD1-synthesis agonists, this survival mechanism might potentiate neurotrophin activity [57]. In addition, it could downregulate cyclooxgenase-2 expression during epileptogenesis [58] and limit neuroinflammatory-mediated damage [19, 56]. We therefore hypothesize that NPD1 attenuates neuroinflammation and upregulates anti-apoptotic signaling in dendrites during epileptogenesis. Although further studies need to be conducted, our studies here show that NPD1 provides effective neuroprotection that down-regulates epileptogenesis and rescues vulnerable cellular and sub-cellular structures after SE. This research could contribute to the development of new therapeutic approaches for circuitry impairment in epileptogenesis, Alzheimer’s and other neurodegenerative diseases.

## Supporting Information

S1 Fig Spontaneous hippocampal interictal spikes in epileptogenesis post-status epilepticus. A: Interictal spike (arrow, IS) from hippocampal local field potentials (LFPs) of a mouse one week post-status epilepticus (PSE). B: Peri-event spectrogram of IS activity. Note that IS (inset) begins in the dentate gyrus (DG) followed by the stratum radiatum (RAD), and then simultaneously in the pyramidal layer (PYR) and stratum oriens (OR) (inset). C: Number of interictal spikes (n = 45) is the same for PSE (n = 7) and control mice (n = 7). Bars represents means and error bars S.E.M P = p values vs. controls t-tests.
(PDF)

S2 Fig Number of dendritic spines between control (n = 3) and NPD1-treated mice (n = 4). Note no significant difference between groups. LM: Lacunosum moleculare DG: Dentate Gyrus. Bars represent means, and error bars S.E.M. P = p values vs. controls t-tests.
(PDF)
