# Supplementary figures and images for: Hippocampal Neuro-Networks and Dendritic Spine Perturbations in Epileptogenesis Are Attenuated by Neuroprotectin D1
Source: PLoS One. 2015 Jan 24;10(1):e0116543. doi: 10.1371/journal.pone.0116543 (PMC4305283; doi:10.1371/journal.pone.0116543)

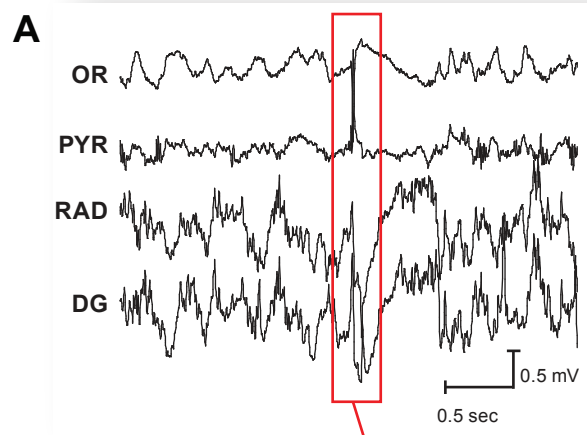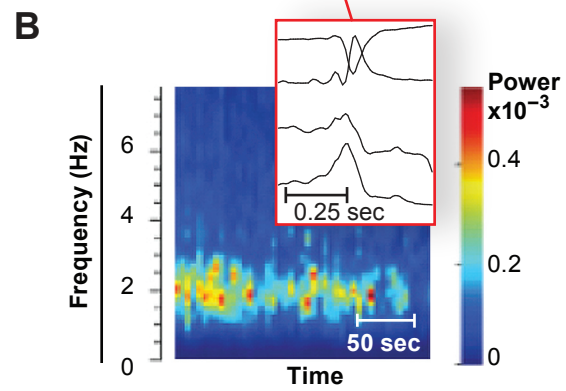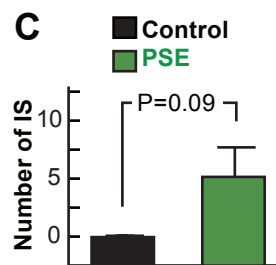

Supplement: S1 Fig — A: Interictal spike (arrow, IS) from hippocampal local field potentials (LFPs) of a mouse one week post-status epilepticus (PSE). B: Peri-event spectrogram of IS activity. Note that IS (inset) begins in the dentate gyrus (DG) followed by the stratum radiatum (RAD), and then simultaneously in the pyramidal layer (PYR) and stratum oriens (OR) (inset). C: Number of interictal spikes (n = 45) is the same for PSE (n = 7) and control mice (n = 7). Bars represents means and error bars S.E.M P = p values vs. controls t-tests. (PDF) [file pone.0116543.s001.pdf]

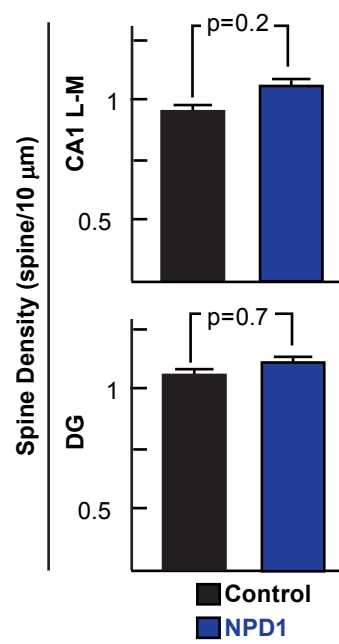

Supplement: S2 Fig — Note no significant difference between groups. LM: Lacunosum moleculare DG: Dentate Gyrus. Bars represent means, and error bars S.E.M. P = p values vs. controls t-tests. (PDF) [file pone.0116543.s002.pdf]
